# Sixteen-Year Longitudinal Evaluation of Blood-Based DNA Methylation Biomarkers for Early Prediction of Alzheimer’s Disease

**DOI:** 10.3233/JAD-230039

**Published:** 2023-08-15

**Authors:** Fernanda Schäfer Hackenhaar, Maria Josefsson, Annelie Nordin Adolfsson, Mattias Landfors, Karolina Kauppi, Tenielle Porter, Lidija Milicic, Simon M. Laws, Magnus Hultdin, Rolf Adolfsson, Sofie Degerman, Sara Pudas, the Australian Imaging Biomarkers and Lifestyle Study

**Affiliations:** a Department of Integrative Medical Biology, Umeå University, Umeå, Sweden; b Umeå Center for Functional Brain Imaging, Umeå University, Umeå, Sweden; c Department of Statistics, USBE, Umeå University, Umeå, Sweden; d Center for Ageing and Demographic Research, Umeå University, Umeå, Sweden; e Department of Clinical Sciences, Umeå University, Umeå, Sweden; f Department of Medical Biosciences, Pathology, Umeå University, Umeå, Sweden; g Centre for Precision Health, Edith Cowan University, Joondalup, WA, Australia; h Collaborative Genomics and Translation Group, School of Medical and Health Sciences, Edith Cowan University, Joondalup, WA, Australia; i Curtin Medical School, Curtin University, Bentley, WA, Australia; j Department of Medical Epidemiology and Biostatistics, Karolinska Institute, Stockholm, Sweden; k Department of Clinical Microbiology, Umeå University, Umeå, Sweden

**Keywords:** Alzheimer’s disease, biomarkers, DNA methylation, epigenomics, longitudinal studies

## Abstract

**Background:**

DNA methylation (DNAm), an epigenetic mark reflecting both inherited and environmental influences, has shown promise for Alzheimer’s disease (AD) prediction.

**Objective:**

Testing long-term predictive ability (>15 years) of existing DNAm-based epigenetic age acceleration (EAA) measures and identifying novel early blood-based DNAm AD-prediction biomarkers.

**Methods:**

EAA measures calculated from Illumina EPIC data from blood were tested with linear mixed-effects models (LMMs) in a longitudinal case-control sample (50 late-onset AD cases; 51 matched controls) with prospective data up to 16 years before clinical onset, and post-onset follow-up. Novel DNAm biomarkers were generated with epigenome-wide LMMs, and Sparse Partial Least Squares Discriminant Analysis applied at pre- (10–16 years), and post-AD-onset time-points.

**Results:**

EAA did not differentiate cases from controls during the follow-up time (*p* > 0.05). Three new DNA biomarkers showed in-sample predictive ability on average 8 years pre-onset, after adjustment for age, sex, and white blood cell proportions (*p*-values: 0.022-<0.00001). Our longitudinally-derived panel replicated nominally (*p* = 0.012) in an external cohort (*n* = 146 cases, 324 controls). However, its effect size and discriminatory accuracy were limited compared to *APOE* *ɛ*4-carriership (OR = 1.38 per 1 SD DNAm score increase versus OR = 13.58 for *ɛ*4-allele carriage; AUCs = 77.2% versus 87.0%). Literature review showed low overlap (*n* = 4) across 3275 AD-associated CpGs from 8 published studies, and no overlap with our identified CpGs.

## INTRODUCTION

Alzheimer’s disease (AD) pathology in the form of tau and amyloid-β (Aβ) starts accumulating years or decades before clinical symptoms [1], making early prediction critical. Recently, novel blood-based markers, for instance those reflecting different species of phosphorylated tau in plasma have shown promise for AD prediction [2, 3]. In addition, the study of polygenic risk-scores (PRS) for AD has come to explain an increasing proportion of AD heritability [4]. However, it is essential to study further non-invasive biomarkers, besides the neuropathology and genetic biomarkers, due to the highly heterogeneous and multifactorial nature of AD, which is influenced by multiple lifestyle and environmental factors across life [5–7].

DNA methylation (DNAm), an epigenetic process measurable in blood, is of increasing interest for AD prediction due to its potential to capture both inherited and acquired disease risk through the life course [8–10]. DNAm is a mechanism that can regulate gene expression, by binding of methyl groups to DNA nucleotides, most commonly at CpG sites (cytosine-guanine nucleotide pairs). DNAm patterns are partially heritable, influenced by lifestyle and environmental factors, and change in aging [9, 11, 12]. Epigenetic biomarkers based on DNAm are also of interest for prediction of biological aging, aging-related diseases, and mortality [13, 14]. Of interest is that DNAm can bring potential mechanistic insights into disease etiology thanks to its potential to affect gene expression [15]. In AD, DNAm alterations located in gene regions with well-established roles in AD have been identified in both brain tissue and blood, including apolipoprotein E (*APOE*) [16] and the amyloid precursor protein (*APP*) genes [17, 18]. Across studies however, limited replication across CpG sites and genes have been found so far, with the direction of some effects differing between studies, i.e., hyper- versus hypomethylation in AD cases [19]. DNAm findings in blood cells can be challenging to interpret in the context of neurodegenerative diseases due to the tissue-specificity of DNAm [20, 21]— although some CpG-sites’ methylation levels show high correspondence across blood and brain [21–23]. In theory, DNAm changes in blood cells of AD patients could be the consequences of disease-related processes, capture potential causal pathways relevant for disease progression such as peripheral inflammation or immune system functioning [14, 24], or could mirror the disease-causal methylation status of the corresponding CpG-site in brain tissue [25, 26]. In all these scenarios, blood DNAm patterns could hold value as biomarkers if they significantly contribute to the prediction of disease conversion or progression.

The relative consistency of DNAm changes with aging enables accurate estimation of chronological age based on DNAm patterns using machine learning methods, resulting in epigenetic age-estimators, or “clocks” [13, 27]. A higher epigenetic age than chronological age as estimated by epigenetic clocks, so-called epigenetic age acceleration (EAA), has been found to associate with cellular and physical aging, mortality [14, 28], several age-related diseases including Parkinson’s disease, some cancer types, coronary heart disease [13, 14] as well as AD [29]. Since advanced age is the largest risk factor for late-onset sporadic AD [30], it is of relevance to investigate whether measures capturing accelerated biological aging are predictive of AD. In studies of AD, EAA in brain tissue correlates with neuropathological biomarkers of AD, including brain tau and amyloid load, as well as with decline in cognition [29, 31, 32]. The fact that AD-related DNAm alterations in brain tissue are enriched in sites displaying aging-related changes, with a concordance of effect direction, also suggests that EAA may be of relevance for AD prediction or disease progression [33]. However, previous studies have found mixed results with regards to the utility of EAA in blood in the context of dementia [8, 34–36], and there is a scarcity of longitudinal studies tracking EAA over longer time-periods preceding disease onset. Thus, although EAA in blood can successfully predict a number of aging-related outcomes, its predictive value for AD remains to be established.

The current study aimed at testing the hypothesis of accelerated epigenetic aging in blood in clinical late-onset AD using a longitudinal case-control design, with up to 20 years of longitudinal follow-up data from the same carefully characterized individuals from a Swedish population-based study [37, 38]. We tested three validated epigenetic clocks thought to capture different aspects of cellular and physiological aging [14, 39, 40], as well as a DNAm biomarker designed to capture the rate of biological aging across multiple organ systems [41, 42]. In addition, we investigated whether we could identify sets of CpG sites predictive of AD up to 16 years before clinical AD onset, using both univariate and multivariate statistical methods. First, using CpG-wise univariate linear mixed effects models (LMMs), we leveraged our longitudinal data to identify CpG sites that stably differentiated AD cases from controls across the entire follow-up period, with the rationale that such sites may be particularly robust biomarkers. Secondly, we used a multivariate supervised machine-learning method for variable selection by sparse partial least squares discriminant analysis (sPLS-DA), which accounts for the covariance among CpG sites, and permits selecting sets of CpGs able to conjunctly discriminate AD cases from controls. By applying the sPLS-DA in specific time subsamples, long before (16-10 years) and after AD onset, we tested whether different sets of CpGs were predictive at different disease stages, substantially expanding the time frame of previous studies [43, 44]. Finally, we tested whether the findings from our primary study cohort replicated in a cross-sectional Australian sample [45], or corresponded to previously reported DNAm differences in AD based on a literature search. An overview of the study design is provided in Supplementary Figure 1.

## MATERIALS AND METHODS

### Study populations

Our main study sample is from the Betula study, a longitudinal, prospective population-based study, aimed at investigating health, aging, cognition and dementia. It comprises 4,425 participants followed for up to 25 years, with cognitive, health-related, social, and biological assessments [37, 38, 46]. As fully described previously [38, 46], the recruited participants were native Swedish speakers with no dementia, congenital or acquired intellectual disabilities at study entry, nor severe impairments in hearing or vision. The study was initiated in 1988 with consecutive follow-ups at five years intervals (T1–T6 test waves), evaluating cognitive status and dementia at each time point. Blood sampling was carried out at test waves T2–T6 (Supplementary Table 1).

The Australian Imaging, Biomarker & Lifestyle Study (AIBL) was used as an independent external cohort to validate the analyses. Data was collected by the AIBL study group. AIBL study methodology has been reported previously [45, 47]. The AIBL study aimed at recruiting and characterizing 1000 participants, including at least 200 AD cases, 100 mild cognitive impairment (MCI) cases, and 700 healthy controls (aimed at including both carriers and non-carriers of the *APOE* *ɛ*4 allele, and subjects with subjective memory complaints). More than 4000 subjects initially volunteered after a media appeal or were informed about the study by a clinician. In all, 1,166 individuals were assessed and screened, resulting in a final baseline sample comprising 768 healthy controls, 133 MCI cases, and 211 AD cases.

This research was conducted in accord with the Declaration of Helsinki. The Betula study has been approved by the Regional Ethical Review Board in Umeå and the Swedish Ethical Review Authority, and written consent for study participation was obtained from each participant. Informed written consent was also given by all AIBL volunteers, and ethics approvals for the study were obtained from ethics committees of Austin Health, St. Vincent’s Health, Hollywood Private Hospital and Edith Cowan University.

### 2.2 Clinical characterization and dementia diagnosis assessments

The dementia diagnosis assessment in the Betula study has previously been described [37]. In brief, the diagnostic characterization was based on multiple sources of information and included each participant’s healthcare history as expressed in medical records, supplemented by relevant data from the repeated health and cognitive assessments that were part of the Betula study protocol. The dementia diagnoses were defined according to the Diagnostic and Statistical Manual of Mental Disorders 4th edition (DSM-IV) dementia classification. Participants diagnosed with AD showed representative symptoms of clinical AD, including an insidious onset and progressive cognitive decline.

For the AIBL sample, AD diagnoses were based on NINCDS-ADRDA Alzheimer’s Criteria (probable or possible), evaluating the impairment of memory, language, perceptual skills, attention, constructive abilities, orientation, problem solving, and functional abilities [48]. MCI diagnosis was based on Winblad et al., in which subjects previously diagnosed with MCI by a clinician additionally showed a score at least 1.5 standard deviation from the age-adjusted mean on one or more neuropsychological tasks [49].

### Inclusion and exclusion criteria

The Betula sample inclusion criteria were having at least one blood sample ≥3 years prior to clinical AD onset, and at least one sample at or after onset, as well as a ≥5-year duration between samples. Only cases with an onset age of ≥65 years were considered. In total, 49 AD cases fulfilled these criteria. An additional two cases without post-onset samples, but with pre-onset samples >10 years prior to onset were added to enrich the sample with measurements collected long before onset. Thus, the final sample comprised 51 AD participants and 51 healthy age- and sex-matched controls subjects, born between 1909 and 1944. This selected sample comprised 20 AD cases and 20 matched controls with three longitudinal time-points, 29 AD cases and 29 matched controls with two longitudinal time-points, and 2 AD cases and 2 matched controls with one time-point long before AD onset. Controls were considered healthy when not diagnosed with AD or any other dementia subtypes, and not showing memory decline according to a previous classification model applied to the sample [50, 51]. The majority of the included controls (*n* = 42) were classified as ‘average memory decline’ according to the classification, the reminder showed above average memory maintenance (*n* = 9), indicating normal/non-pathological memory aging among controls. In total, 237 blood samples (AD + controls) were analyzed on DNAm arrays. Among those, four samples were excluded due to mismatch or not passing the built-in quality control test in the arrays. Thus, 233 samples remained for further analyses, comprising 50 AD cases (116 samples) and 51 matched controls (117 samples), with a median of 16 years (min 5, max 26) of follow up time.

The AIBL DNAm sample comprised 471 healthy controls, 94 MCI cases, and 161 AD cases (see sample demographic characteristics in Supplementary Table 2). We excluded subjects younger than 65 years of age (*n* = 95) as the focus was on late-onset clinical AD, and the same age-range criteria were used for the controls and the MCI group. Healthy controls that developed AD (*n* = 9) or MCI after the DNA methylation analysis (*n* = 64) were also excluded. Thus, 324 healthy controls, 88 MCI cases, and 146 AD cases were included (Supplementary Table 2).

### DNAm analyses

#### DNA bisulfite conversion and methylation array analysis

DNA was extracted from peripheral blood (whole blood or buffy coat) collected at multiple blood sampling time points, by previously described methods in Betula [52] and AIBL [53]. In Betula, the DNA were sodium bisulfite converted using the EZ DNA methylation kit (Zymo Research, CA, USA) according to the manufacturer’s protocol. AIBL DNA methylation was obtained from the NCBI Gene Expression Omnibus GSE153712 [54]. Infinium Methylation EPIC BeadChip arrays (Illumina inc., San Diego, CA) were used in Betula and AIBL for methylation profiling of the bisulfite converted DNA. These arrays interrogate over 850 000 CpG sites across the genome at single-nucleotide resolution. For the Betula cohort the quality of the methylation data was assessed using the bead arrays controls reporter (Illumina) and the multiple samples from the same individual were confirmed using single-nucleotide polymorphisms (SNPs) included on the array. In both Betula and AIBL arrays, raw methylation data from the arrays (β-values) was extracted using the the *minfi* R package and were normalized using the BMIQ (Beta MIxture Quantile dilation) normalization (V1.3). The average beta (avg. β) methylation level of each CpG site ranges from 0 (unmethylated) to 1 (fully methylated). Probes with detection *p*-values greater than 0.05 were set as missing values prior to normalization. Multimapping probes, and probes where the methylation site is close to a SNPs in European population were filtered out based on previous recommendations [55]. Methylation sites known to be under the influence of DNA sequence variants and associated with, e.g., ethnicity (methylation quantitative trait loci, meQTLs) in *cis* or in *trans* [56, 57], and methylation sites in the X and Y chromosomes were also filtered out prior to analysis. Batch effects were assessed, and no batch effect correction was deemed necessary. After these filtering steps, 690,926 CpG sites remained for analysis. In addition, for the univariate and multivariate analyses, 5,412 probes with any missing value were excluded, totaling 685,514 CpG sites remaining for analyses.

#### Epigenetic clocks estimation

Hannum’s DNAm age (71 CpGs) was originally calculated composing a weighted average (formed by regression coefficients) of CpGs, which then is transformed to DNAm age using a calibration function [39]. Horvath’s epigenetic clock (353 CpGs) is based on a similar regression model approach [40]. Hannum’s and Horvath’s clocks were constructed with the Illumina 450k methylation array, with 6 and 17 included CpGs, respectively, missing on the currently used Methylation EPIC array [13]. The PhenoAge clock (513 CpGs) was obtained by a penalized regression model that accounted for several disease risk biomarkers [14]. All CpGs used by the PhenoAge clock are available on the EPIC array. We also estimated the DNAm biomarker Dunedin Pace of Aging Calculated from the Epigenome (DunedinPACE) [41], an updated version of the DunedinPoAm clock designed to predict the longitudinal rate of change in 18 biomarkers from multiple organ systems across 12 years [42]. For the epigenetic clocks estimation, missing β-values were imputed by a K-nearest neighbor model.

To estimate epigenetic age acceleration/deceleration, delta epigenetic ages were used instead of age-acceleration residuals from linear regression-based estimation, since the longitudinal measures violate the assumption of independence of observations (see also [58]). Thus, after the estimation of Horvath, Hannum, and PhenoAge epigenetic age clocks, Δepigenetic ages were obtained by subtracting the chronological age from the epigenetic age. A positive Δepigenetic age indicates accelerated epigenetic aging (i.e., the individual is biologically older than their chronological age) and a negative Δepigenetic age indicates slower epigenetic aging (i.e., an individual is biologically younger than their chronological age). The raw estimated values were used for the DunedinPACE health clock, as it is not an age estimator. One healthy control subject presented values below 3 standard deviations from the mean Hannum, Horvath and PhenoAge clocks, and these outlier values were replaced by the second lowest value of the full sample to avoid exclusion and loss of data points, according to a previous study [59].

### Covariates

For the Betula sample*, APOE* genotypes were obtained by polymerase chain reaction (PCR), as previously described [60]. *APOE* was set as a binary indicator variable (0/1), indicating the absence/presence of an *ɛ*4 allele. The blood cell proportions of granulocytes, cluster of differentiation (CD)8+ T-cells, CD4+ T-cells, Natural Killer (NK) cells, B cells, and monocytes were estimated from the DNAm array [61]. Granulocyte proportion was chosen to adjust the models, as it is the estimated blood cell type with the highest proportion in the blood, which may contribute to the DNAm levels reflected by the DNAm array [62]. In the analyses, the considered health- and lifestyle covariates from each subjects’ baseline were body mass index (BMI), waist-hip-ratio, blood glucose, erythrocyte sedimentation rate, pulse pressure, years of education, and a binary indicator of whether the participant ever reported to be a smoker during the study period (Supplementary Table 1). Backward selection was used to select covariates to be included in the ΔDNAm clocks adjusted LMMs. However, except for the self-reported smoking indicator, these covariates showed no associations with the DNAm clocks in preliminary LMMs, and no significant differences between AD cases and matched-controls (descriptives in Supplementary Tables 1 and 2). In the novel DNAm biomarkers’ logistic and Cox models, none of the lifestyle-related markers were significant; therefore, they were not included in the final regression models. Carriage of the *APOE* *ɛ*4 allele, sex, self-reported smoking, and AD status were included in the models as binary indicator variables (0/1). In the AIBL sample, the variables AD status, age at AD and MCI onset, *APOE*, sex, granulocyte proportion, years of education, self-reported smoking, Hannum, Horvath, PhenoAge, DunedinPACE, and chronological ages were set as described for the Betula sample.

Relative leukocyte telomere length (RTL) was compared to the novel DNAm biomarkers, as an established blood-based biomarker previously associated with increased AD incidence in non-*APOE* *ɛ*4-carriers in our sample [63]. Peripheral blood leukocytes DNA was used to estimate normalized RTL as previously described [52, 63], using a modified Cawthon’s polymerase chain reaction method [64, 65]. Preliminary visual inspection of our data indicated a potential differential RTL attrition between cases and controls. To capture the attrition of RTL over the study period, individual RTL slopes were estimated as the beta-coefficients from linear models of RTL predicted by the age at RTLsampling.

### Statistical analyses

#### Generalized additive mixed models (GAMMs)

GAMMs were used to depict the longitudinal profile of the raw estimated DNAm clocks in cases and controls separately, in order to check for potential non-linear associations in the Betula sample. Subsequent analyses used linear models, as observed associations were highly linear. Unadjusted GAMM models were performed in R using the *gamm4* function of the *gamm4* package.

#### Linear mixed-effects models

In the Betula sample, longitudinal changes in the DNAm biomarkers (Hannum, Horvath, PhenoAge, or DunedinPACE), and blood cell proportions were assessed employing LMMs. As we intended to access differential longitudinal changes between AD cases and controls, all LMMs include an interaction term between AD status as a binary indicator variable and time, calculated in years to clinical onset (year 0). The time-scale ranged from – 16 to 7, i.e., a 23-year follow-up duration was modelled. Chronological age was used as an alternative time-scale. The longitudinal measures from the same subject and the sex- and age-matched pairs were modelled as nested random effects to account for variability within these blocking variables, therefore there was no need to control the models for age and sex. In particular, subjects were nested within matched pairs such that each matched pair of subjects are unique to that pair. LMMs were performed in R using the *lmer* function of the *lme4* package. Thus, the models were set as: lmer(DNAm biomarker ∼ *APOE* *ɛ*4 carrier + ever smoked + granulocyte proportion + AD status × time to/after AD onset (or chronological age) + (1 | subject number) + (1 | matched pair number). Detailed R codes of the GAMMs and LMMs are available at https://fernandashackenhaar.github.io/LMMs/.

#### Univariate longitudinal analysis of differentially methylated sites

We used LMMs with individual CpG-sites as dependent variables to identify differentially methylated sites that stably discriminate AD cases from controls across the entire follow-up period (see the statistical analysis section above for a more detailed description of the LMMs). Models were adjusted for *APOE* *ɛ*4 carriage and granulocyte proportion. No CpG passed the 5% cut-off for Benjamini– Hochberg false discover rate (FDR) correction. Instead, an exploratory approach was used where CpGs were selected when having a significant (*p* < 0.001) model estimate (beta coefficients) for AD of at least 0.05 (≥|0.05|). As the CpGs’ β-values range from 0 to 1, an estimate of |0.05| represents a methylation difference between AD cases and controls of 5%. CpGs were further required to show absence of a cross-over interactions between AD status and time, in other words, that the direction of the association remained the same before and after AD onset [66, 67] (see the beta-values of a representative CpG selected by this method in the Supplementary Figure 2). The β-values of the CpGs that passed these criteria were multiplied by 1 or –1 according to the direction of association with AD, and summed up to obtain the marker hereafter denoted *The longitudinal AD panel* (see the CpGs and their directions of association in Supplementary Table 3). As a *post-hoc* analysis we also tested a version of the panel where CpGs were weighted with the effect sizes (beta coefficients) from the LMM, but the weighted longitudinal AD panel did not outperform the original longitudinal AD panel (data not shown).

As a sensitivity analysis, we additionally ran the LMMs on 78 filtered out meQTL sites that have previously been associated with AD [56, 57]. None of the sites fulfilled the *p* < 0.001 threshold (*p*-values = 0.009–0.980) and the |Δβs| were lower than 3.8% and would thus not have been selected into our panel.

#### Multivariate analysis of AD-predicting sets of CpGs

Machine learning-based sparse partial least squares discriminant analysis (sPLS-DA) [68] with 685,514 CpGs was used to identify CpGs that together may differentiate AD from controls. Two different cross-sectional subsamples were used for the sPLS-DA analyses: 1) the ‘long before’ AD subsample, that comprised samples from 16 to 10 years before AD onset and their respective matched controls (21 AD cases and 19 controls); and 2) the ‘after AD’ subsample, comprising samples from the years of AD onset to 7 years after AD onset (47 AD cases and 49 controls). sPLS-DA combines variable selection (identifying the most predictive or discriminative CpGs using lasso penalization) and classification in a one-step procedure. The algorithm uses a linear transformation that converts the data into a reduced dimensional space, in which the principal components (PCs) are the estimated features that represent the reduced dimensions that best separate the labeled groups with the smallest error rate. The number of PCs and CpGs within the PCs was selected by the lowest obtained balanced error rate (BER) after within-sample cross-validation (3-fold repeated 50 times). The sPLS-DA analyses were performed by the *splsda, tune.splsda*, and *perf* functions of the *mixOmics R* package. To obtain AD predictive scores for each subject, the β-values of the identified CpGs were multiplied by the weight (loading) of each DNAm site obtained by the sPLS-DA analysis, and thereafter summed. The loadings also indicate the direction of the DNAm site association with AD. Positive loadings indicate CpGs that are hypermethylated in the AD cases, and negative loadings indicate CpGs that are hypermethylated in the healthy matched controls.

#### Logistic regression models

The ability of each DNAm clock and novel DNAm biomarker to differentiate AD cases from controls was evaluated in the Betula sample at baseline time-point of each participant on average 8 years before AD onset, by logistic regressions. Models were adjusted for the covariates *APOE* *ɛ*4 allele carriage, granulocyte proportion, sex, and chronological age. In these models, and the ones described below, DNAm clocks, novel DNAm biomarkers, age, and granulocyte proportion were z-transformed, i.e., scaled to zero-mean and standard deviation (SD) of one. Thus, effect sizes should be interpreted as reflecting one standard deviation’s increase in the odds/hazard ratio. Self-reported smoking was not significant in these models, thus not shown. Binary logistic regressions were performed in R using *glm* function of the *stats* package.

#### Cox proportional hazard regression models

The ability of each DNAm clock and novel DNAm biomarkers to predict the risk of AD was evaluated at baseline time-point of each participant, on average 8 years before AD onset, by Cox regression. Models were adjusted for the confounders *APOE* *ɛ*4 allele carriage, granulocyte proportion, sex, and chronological age. Cox regressions were performed in R using the *coxph* function of the *survival* package. Due to collinearity between some of the DNAm clocks and novel DNAm biomarkers (see correlation Supplementary Table 4), the predictors could not all be included in a single model [69]. For this reason, Cox and logistic models were estimated for each DNAm biomarker separately.

#### Internal validation analyses

C-statistics of the logistic regression models were used to compare the discriminatory accuracy of the novel DNAm biomarkers estimated in Betula with the established biomarker *APOE* *ɛ*4 allele carriage, at baseline time-point of each participant, on average 8 years before AD onset. The models were adjusted for the confounders granulocyte proportion, sex, and chronological age. C-statistics forest plot was estimated using the *forestplot* function of the *forestplot* package in R.

#### External validation analyses

Logistic regressions and their respective area under the receiver operating characteristic (ROC) curves AUC evaluating models’ discriminatory accuracy were used to validate the novel DNAm biomarkers in the AIBL validation sample. As for Betula, logistic models were adjusted for *APOE* *ɛ*4 allele carriage, granulocyte proportion, sex, and chronological age. ROC curves were estimated using the *roc* function of the *pROC* package in R.

#### Enrichment analysis

Tests of enrichment of CpGs-associated genes in AD or AD-related pathways by ‘Pathway’, ‘Disease’, and ‘Human Phenotype’ was performed by protein function analysis using Toppgene (https://toppgene.cchmc.org/enrichment.jsp). Enrichment by ‘disease biomarker networks’, and ‘diseases (by biomarkers)’ was performed with GeneGo MetaCore^TM^ software (https://portal.genego.com/).

## RESULTS

### Sample characteristics

The Betula and AIBL samples selected for the DNAm analysis were compared to investigate potential sample differences (Table 1). The percentage of males was similar between cases and controls within the samples, but higher in AIBL (∼43%) than in Betula cases (∼18%) (Table 1). In Betula, the proportion of *APOE* *ɛ*4-carriers among AD cases (54%) and matched-controls (19.6%) were also similar to the proportions in the full population-based samples (Supplementary Table 1), indicating the representativeness of our selected study sample. In both samples, there is an expected higher proportion of *APOE* *ɛ*4-carriers in AD cases than controls (Table 1), although higher proportions were seen in AIBL compared to Betula (due to enrichment for APOE *ɛ*4 at sampling). The age at clinical onset was on average 81 years (range 67–94 years) in Betula and 78.5 in the AIBL sample (Table 1). While in the AIBL sample there were age-differences between controls (∼73 years), MCI (∼77 years), and AD cases (∼80 years), the Betula sample cases and controls were age-matched (∼75 years at study enrollment). For a graphical description of the Betula longitudinal blood sampling times in relation to each participant’s chronological age and age at AD onset, seeFig. 1.

**Table 1:** Demographic characteristics of the Betula and AIBL study populations

	Betula study participants		AIBL study participants		
	Controls (*n* = 51)	AD (*n* = 50)	Controls (*n* = 324)	MCI (*n* = 88)	AD (*n* = 146)
Sex, female/male (%)	42 / 9 (17.6%)	41 / 9 (18%)	184 / 140 (43%)	33 / 55 (63%)	82 / 64 (43%)
*APOE* *ɛ*4-carriers, *n* (%)	10 (19.6%)	27 (54%)	76 (23%)	41 (47%)	112 (77%)
Age at AD onset, y	–	81 (67–94)	-	-	78.5 (65–93)
Age, y*	75 (55–86)	75 (55–85)	73 (65–91)	77 (66–95)	80 (66–93)
Years of education					
0 to 6	23 (45.1%)	18 (36%)	1 (0.31%)	1 (1.14%)	9 (6.16%)
7 to 8	11 (21.57%)	17 (34%)	22 (6.79%)	10 (11.36%)	20 (13.71%)
9 to 12	11 (21.57%)	8 (16%)	125 (38.58%)	39 (44.32%)	51 (34.93%)
13 to 15	4 (7.84%)	5 (10%)	61 (18.83%)	15 (17.04%)	31 (21.23%)
15 or more	2 (3.92%)	2 (4%)	114 (35.18%)	23 (26.14%)	32 (21.92%)
missing	0 (0%)	0 (0%)	1 (0.31%)	0 (0%)	3 (2.05%)

Data are expressed as number (percentage) or median (min-max). AD, Alzheimer’s disease. *APOE*, apolipoprotein E. MCI, mild cognitive impairment. ^ *^Age at study entry for Betula, and age at blood sampling for AIBL.

**Fig. 1:**
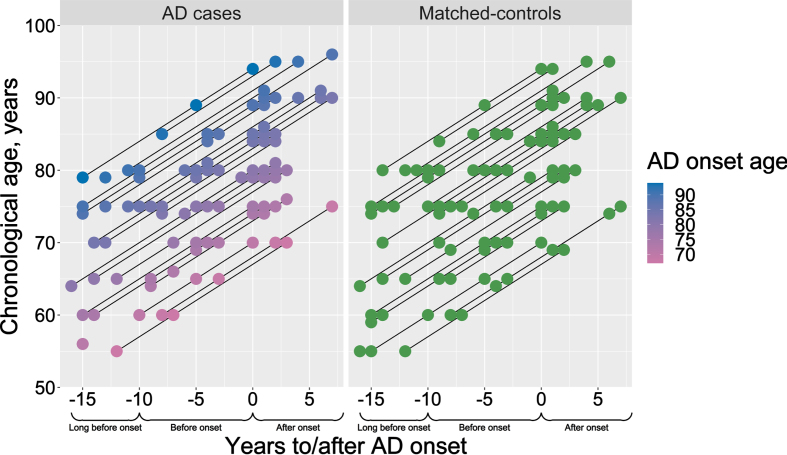
Description of the study design. Chronological age and AD onset age of the selected AD cases (pink-to-blue) and their respective sex- and age-matched controls (green). Y-axis presents the participants’ chronological age at blood sampling, and the color scale bar represents the age of AD onset. X-axis represents the time-scale, where blood samples for the DNAm analysis were selected aiming three time-points: long before (–16 to –10 years before AD onset), before (–9 to –3 years before AD onset) and after AD onset (0 to 7 years after AD onset).

In Betula, among health and lifestyle variables, BMI was significantly lower in the AD cases after clinical onset but the remaining of the considered covariates did not differ between the study groups at baseline (Supplementary Table 1).

### Longitudinal changes in blood cell proportions

Prior to biomarker analyses, LMMs were used to evaluate whether aging or AD were associated with longitudinal changes in estimated blood cells proportions (Supplementary Table 5 and Supplementary Figure 3) in the Betula sample. The AD cases had a faster rate of increase in the NK cell proportion associated with increased chronological age (interaction beta coefficient = 0.0006, *p* = 0.047). We found no other significant differences in the intercepts or the slopes of the other blood cells, indicating similar blood cell proportions in AD cases and matched-controls. CD4+ T cells and B cells had decreased proportions associated with increase in chronological age (i.e., significant negative slopes, Supplementary Table 5). No significant associations were found for the CD8+ T cells, while granulocytes and monocytes had increased proportions associated with increase in chronological age (i.e., significant positive slopes, see Supplementary Table 5) (Supplementary Figure 3d). As a few CpG sites within a gene region may be hypomethylated in one cell type but be hypermethylated on another, the cell type with the biggest proportion will affect the overall methylation pattern, so the cell composition can affect the investigated DNAm patterns in blood [62]. On average, the proportion of granulocyte was higher (60%) than all the other DNAm-estimated blood cell types (CD8+ T cells = 8%, CD4+ T cells = 14%, NK cells = 6%, B cells = 4%, monocytes = 8%), therefore it was used to adjust subsequent analyses.

### 3.2 AD cases are not epigenetically older than healthy sex- and age-matched controls

**Fig. 2:**
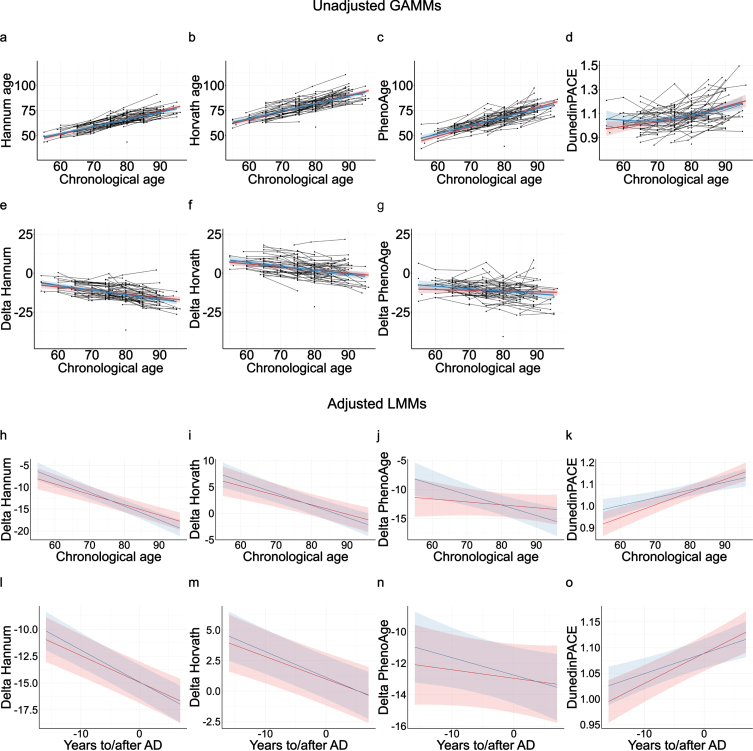
Longitudinal analyses of DNAm clocks in AD cases (red) and matched-controls (blue). Unadjusted generalized additive mixed models (GAMMs) of the raw estimated DNAm clocks (a–d) and Δages (e–g) with chronological age as the time-scale. Note negative association between Δage and age, indicating a deacceleration of epigenetic ages at higher chronological age for both cases and controls. Linear mixed-effects models (LMMs) of the Δages and the DunedinPACE clock with chronological age (h–k) and time to/after AD onset (l–o) as time-scales. LMMs were adjusted by *APOE* *ɛ*4 allele carriage, self-reported smoking, granulocyte proportion, and included an Alzheimer’s disease (AD) status by time interaction.

In Betula, the estimated Hannum (Fig. 2a), Horvath (Fig. 2b), and PhenoAge (Fig. 2c) DNAm age clocks showed high and approximately linear associations with chronological age at blood sampling (*rs* = 0.725–0.783; Supplementary Table 4). As expected, the DunedinPACE clock showed a less clear association with chronological age (Fig. 2d). Unadjusted LMMs with chronological age as time scale showed no significant differences in the intercepts or the slopes of the DNAm clocks between AD cases and matched controls (*p* > 0.05; Supplementary Table 6).

Delta ages were used to capture EAA, i.e., whether subjects were epigenetic older or younger than expected from their chronological ages. ΔHannum age (Fig. 2e), ΔHorvath age (Fig. 2f), and ΔPhenoAge (Fig. 2g) did not differ significantly between AD cases and controls in the unadjusted LMMs, and no evidence was obtained for differential longitudinal rates of epigenetic aging between cases and controls (*p* > 0.05; Supplementary Table 6). As in previous literature, all Δages were negatively associated with chronological age [70, 71], indicating a deacceleration of EAA at higher chronological ages. Δages were not associated with *APOE* *ɛ*4 allele carriage (Supplementary Table 7).

LMMs adjusted for additional covariates, *APOE* *ɛ*4-carriage, granulocyte proportion, and self-reported smoking, were also used to assess potential differential longitudinal changes in ΔHannum age, ΔHorvath age, ΔPhenoAge, and DunedinPACE clock between AD cases and controls (Supplementary Tables 7 and 8). Independently of the used time-scale, chronological age (Fig. 2h-k, Supplementary Table 7) or time to/after AD onset (Fig. 2l-o, Supplementary Table 8), the adjusted models did not show any significant differences in DNAm age markers between AD cases and matched controls. If anything, AD cases trended towards having epigenetically younger ΔPhenoAge and a slower Dunedin PACE age at younger ages and earlier time-points before AD onset (Supplementary Table 7). Thus, we did not observe any evidence for AD cases showing accelerated epigenetic aging in blood, but rather a trend for the opposite.

We additionally ran a supplementary set of analyses with age at AD onset instead of chronological age as a covariate to test for potential differences driven by age of onset of the cases. However, due to the sampling scheme in this study, which prioritized cases with a long follow-up time (i.e., available blood samples long before AD onset), chronological age at blood-sampling was highly correlated with age at onset (*r* = 0.841; *p* < 2.2E-16), based on the baseline time-point of each participant, see also Fig. 1). Results from these age-of-onset analyses are shown in Supplementary Table 9 and Supplementary Figure 4 and resemble those with chronological age as the time scale, with cases with *younger* age of onset being estimated as epigenetically younger for ΔHannum and ΔPhenoAge, and thus does not support the hypothesis of AD being associated with accelerated epigenetic aging in blood.

When estimated in the AIBL cohort, the DNAm clocks were in accordance with the null findings in the Betula sample, none of the estimated delta ages nor the DunedinPACE clock were able to significantly discriminate MCI (*p*-values: 0.17–0.87, Supplementary Table 10) or AD cases (*p*-values: 0.25–0.99, Supplementary Table 11) from healthy controls in unadjusted logistic regression models, or in models adjusted for *APOE* *ɛ*4 carriage, sex, granulocyte proportion, and chronological age.

### Longitudinal AD panel of differentially methylated sites is predictive of AD 8 years before clinical onset

In the Betula sample, univariate LMMs were used to identify differentially methylated sites that significantly discriminated AD cases from controls longitudinally, i.e., across the study duration of 20 years. There was no indication of inflation, as suggested by the obtained genomic inflation factor (lambda) of 1.050. No CpGs survived FDR-correction, but the models identified 73 CpG sites that fulfilled our exploratory criteria of *p* < 0.001, beta coefficient≥|0.05|, and absence of crossover interaction with time (Supplementary Table 3). These CpGs were thereafter used to compose a longitudinal AD panel score (for violin plots of distribution, see Supplementary Figure 5a). In models additionally adjusted for *APOE* *ɛ*4 carriage, sex, granulocyte proportion, and chronological age, this *longitudinal AD panel* was able to discriminate AD cases and matched-controls (see logistic regression in Supplementary Table 12) and significantly predict the risk of AD (see Cox regression in Supplementary Table 13) at the baseline time-point of each participant, on average 8 years before clinical onset.

Further characterization of the 73 identified sites showed that the median difference in methylation β-values between cases and controls was 4.4% (min 2.6%, max 20.3%) at baseline, and 6.1% (3.6–17.6%) after AD onset (only sites with beta coefficient≥|0.05| at AD onset, see Methods). A majority of the identified sites (57 of 73) were hypomethylated in the AD cases when compared with the controls, and from the 27 CpGs that had significant AD-by-time interactions, all but one had a negative coefficient, i.e., almost all had decreased methylation over time in AD (Supplementary Table 3). Moreover, 22 CpGs had significant longitudinal aging effect (i.e., change over time across the whole sample), of which 19 (86% of 22 CpGs) showed an increase in methylation over time (i.e., positive beta coefficients, but with low effect sizes).

Complementary enrichment analyses of the annotated genes associated with the longitudinal AD panel’s CpGs were performed. These analyses did not show significant enrichment in AD using GeneGo (‘Alzheimer disease core network’ false discovery rate (FDR) >0.1, and ‘Alzheimer disease, late onset’ FDR = 0.084) or AD-, neurodegeneration-, or inflammation-related pathways using Toppgene (FDRs >0.1).

### Pre- and post-AD scores from sPLS-DA are predictive of AD 8 years before clinical onset

Multivariate analysis by sPLS-DA was used to select CpGs that significantly discriminate AD cases from matched-controls cross-sectionally, at two different time-points. Two sets of PCs were identified in the *long before AD* (21 AD cases and 19 controls) and in the *after AD onset* (47 AD cases and 49 controls) subsamples. A unique PC of 7 CpGs (Supplementary Table 3) was selected from the long before AD onset subsample, while two PCs with 2 and 25 CpGs (Supplementary Table 3) were selected from the after AD onset subsample. We estimated weighted scores from these PCs, denoted the *pre-AD score* (for violin plots, see Supplementary Figure 5b) and *post-AD PC1 and PC2 scores* (Supplementary Figure 5c and d, respectively). Using models additionally adjusted for *APOE* *ɛ*4 carriage, sex, granulocyte proportion, and chronological age, we verified that the pre-AD score was significantly able to discriminate AD cases and matched-controls (see logistic regression Supplementary Table 12), and was further able to predict the risk of AD (see Cox regression on Supplementary Table 13) at the baseline time-point of each participant of the Betula sample, on average 8 years before onset. However, note that this analysis is partially circular as 42% of the samples (21 cases, 19 controls) was used both for training, and for testing. We further verified that the post-AD PC1 and PC2 scores were both significantly able to discriminate between AD cases and matched-controls (see logistic regression Supplementary Table 12), able to predict the risk of AD (see Cox regression on Supplementary Table 13) at baseline. Thus, the post-AD PC scores trained on DNAm patterns from the time-point after diagnosis were also predictive when estimated from blood drawn at another time-point, on average 8 years before.

Similar to the longitudinal AD panel, the DNA sites of the pre- and post-AD scores were predominantly hypomethylated in the AD cases (Supplementary Table 3). The median |Δ β| of the pre-AD score CpGs was 1.9% (min 1.3%, max 10.4%) long before AD onset. In CpGs of the post-AD scores, the median |Δ β| was 1.4% (min 0.3%, max 8.4%) after AD onset. In addition, 3 CpGs overlapped between the longitudinal AD panel and the post-AD scores, the cg03688665 in the gene body/promoter region of the mitogen-activated protein kinase 4 (*MAPK4)*, and cg23379980 and cg12934659 outside annotated gene regions (Supplementary Table 3). Thus, although there is some convergence between the univariate and multivariate analysis approaches, they largely identify separate CpGs.

Enrichment analyses of the annotated genes associated with pre- and post-AD scores CpGs were performed and did not show significant enrichment in AD-associated genes using GeneGo (‘Alzheimer disease core network’ FDR >0.1, and ‘Alzheimer disease, late onset’ FDRs >0.1) or AD-, neurodegeneration-, or inflammation-related pathways using Toppgene (FDRs >0.1).

Finally, we also tested whether the novel DNAm panels and scores, measured at study baseline, explained unique variance when considered simultaneously in a Cox regression, along with age, sex, granulocyte proportion, *APOE*, as well as the RTL slope as a comparison biomarker. The longitudinal AD panel and the post-AD score PC2 were significant over and above other predictors (Supplementary Table 14). The correlations between our novel panels and scores, the epigenetic clocks, granulocyte proportion, chronological age, and sex differences are reported descriptively in Supplementary Table 4.

### Internal validation analyses of the novel DNAm biomarkers

**Fig. 3:**
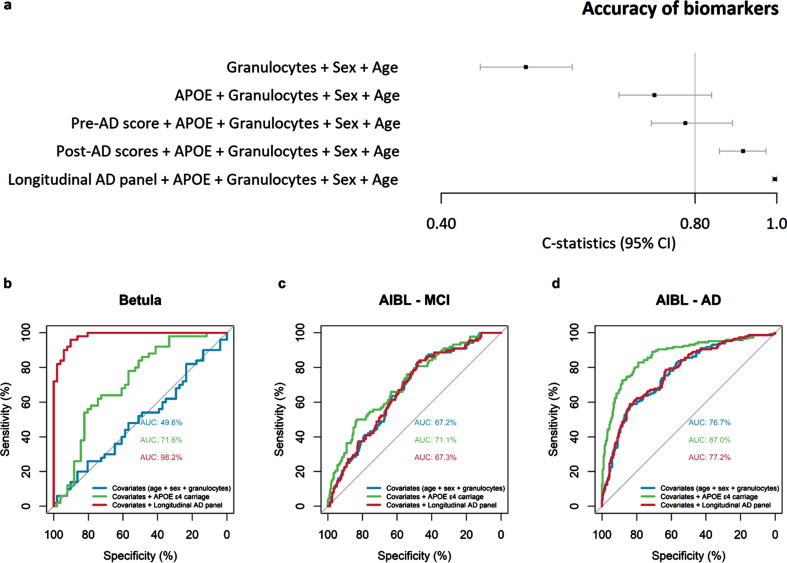
Accuracy of novel DNAm biomarkers in the Betula and The Australian Imaging, Biomarker & Lifestyle of Ageing (AIBL) samples. The forest plot shows the discriminatory accuracy (C-statistics) of the logistic regression models comparing AD cases versus matched-controls when including the biomarkers apolipoprotein E (*APOE*) *ɛ*4 allele carriage, pre-AD score, post-AD scores and longitudinal AD panel in the Betula baseline subsample, on average 8 years before diagnosis (a). Area under the receiver operating characteristic (ROC) curves (AUC) plots of logistic regression models comparing the accuracy of *APOE* *ɛ*4 allele carriage with the longitudinal AD panel in the Betula at the baseline time-point of each participant, on average 8 years before onset (b), AIBL MCI cases (c), and AIBL AD cases (d). Models were adjusted by the covariates chronological age, sex, and granulocyte proportion. *APOE* *ɛ*4 carriage, and sex is a binary term; the novel DNAm biomarkers, age and granulocyte proportion are continuous and z-transformed. Binary terms as AD, are interpreted as the difference between AD and control when all the other covariates are 0. Z-transformed terms are interpreted as the effect of one standard deviation’s increase in the odds ratio.

The DNAm biomarkers estimated from the baseline time-point of each participant, on average 8 years before onset, were compared in an internal validation analysis to test their discriminatory accuracy using C-statistics. Including the well-established biomarker *APOE* *ɛ*4 allele carriage in the logistic model improved model’s AD discriminatory accuracy from 0.50 to 0.72, while including the pre- and post-AD scores improved model’s accuracy to 0.78 and 0.91, respectively (Fig. 3a). Including the longitudinal AD panel further improved the discriminatory accuracy to 0.99, over and above age, sex, granulocyte proportion, and *APOE* *ɛ*4-carriage (see C-statistics Forest plot Fig. 3a). Thus, the panel and scores demonstrated good discriminatory accuracy, although the C-statistics may have been inflated due to circularity in these analyses, particularly for the longitudinal panel.

### External validation analyses of the novel DNAm biomarkers

Next, we estimated the novel DNAm biomarkers in the AIBL sample among MCI cases (violin plots in Supplementary Figure 6) and AD cases (Supplementary Figure 7), and employed equivalent logistic regression models to compare their discriminatory ability between the Betula and the AIBL samples (Supplementary Tables 10 and 11). The novel DNAm biomarkers did not significantly discriminate MCI cases from controls (odds ratio [OR]: 0.98–1.16, *p*-values: 0.28–0.87), while *APOE* *ɛ*4 allele carriage could discriminate both MCI (ORs: 3.10–3.30, *p*-values: 0.00001–0.00002, Supplementary Table 10) and AD (ORs: 12.27–13.58, *p* < 0.00001) in the AIBL sample. Accordingly, AUC plots showed no improvement of the discriminatory accuracy for MCI or AD when including the pre-AD score, or post-AD PC1 and PC2 scores (Supplementary Figure 8). In the logistic regression models, the longitudinal AD panel could significantly discriminate AD cases from controls (OR = 1.38, *p* = 0.012, Supplementary Table 11), in a model adjusted for age, sex, granulocyte proportion and *APOE*. The obtained OR, reflecting a 38% increase in odds for AD for one standard deviation increase in the longitudinal panel score, was however modest when compared to *APOE* *ɛ*4 allele carriage (OR = 13.85, *p* < 0.00001, Supplementary Table 11). In subsequent analyses, we focused only on the longitudinal AD panel.

The discriminatory accuracy of the longitudinal AD panel was lower in the AIBL sample (see AUC Fig. 3c, d) compared to the Betula sample (Fig. 3b). In the Betula baseline subsample, on average 8 years before onset, including *APOE* *ɛ*4 allele carriage in the logistic model improved the AD discriminatory accuracy (i.e., AUC) from 49.6% to 71.6%, while the longitudinal AD panel improved model’s discriminatory accuracy to 98.2% (Fig. 3b). In the AIBL sample MCI cases, *APOE* *ɛ*4 allele carriage had low discriminatory accuracy, improving the AUC from 67.2% to 71.1%, while the longitudinal AD panel maintained a similar discriminatory accuracy of 67.3% as the model with only age, sex, and granulocyte proportion, AUC = 67.2% (Fig. 3c). In the AIBL AD cases, including *APOE* *ɛ*4 allele carriage in the logistic model improved the AD discriminatory accuracy from 76.7% to 87.0%, while the longitudinal AD panel maintained a similar discriminatory accuracy as the model with only age, sex, and granulocyte proportion AUC = 77.2% versus 76.7% (Fig. 3d). Thus, although significantly replicated in the AIBL sample, the out-of-sample discriminatory accuracy of our longitudinal panel was limited in comparison to the established genetic marker *APOE* *ɛ*4.

We next explored whether the individual CpGs obtained in the longitudinal AD panel from the Betula univariate LMMs were associated with MCI and/or AD in the AIBL sample. A univariate logistic regression analysis was implemented, adjusted for *APOE* *ɛ*4 carriage, sex, granulocyte proportion, and chronological age (Supplementary Table 3). From the 73 sites in the panel, 53 showed the same direction of association in Betula and AIBL. Only three of these sites, showed nominally significant (*p* < 0.05) discriminatory accuracy separating AD cases from controls, being hypomethylated in both Betula and AIBL samples (Supplementary Table 3). These were cg05470393 (OR = 0.75, *p* = 0.026), associated with the promoter region of the gene Inducible T Cell Costimulator – *ICOS*), cg08082436 (OR = 0.69, *p* = 0.008, associated with the promoter region of the gene RB Binding Protein 6, Ubiquitin Ligase – *RBBP6*), and cg11015557 (OR = 0.72, *p* = 0.033, in the gene body of the gene DNA Polymerase Epsilon Catalytic Subunit A – *POLE*). Moreover, cg08082436 could significantly discriminate MCI cases from controls with similar effect sizes (OR = 0.77, *p* = 0.041) compared to the AD model, but this was not the case for cg05470393 (OR = 0.95, *p* = 0.721) or cg11015557 (OR = 0.76, *p* = 0.071).

### Previously identified differentially methylated sites overlap poorly across studies

We additionally conducted a literature search of studies published until August 2022 to investigate whether the CpGs of the longitudinal AD panel were reported in previous array-based epigenome-wide association study (EWAS) studies reporting a list of AD-associated CpGs in whole peripheral blood or white blood cells [10, 43, 72–76]. However, no DNAm site selected by our analyses was previously reported (Supplementary Table 15). We also note that among these previous studies reporting several CpGs associated with AD exclusively in blood, only a few reported overlapping CpGs (Supplementary Table 15). From the 1000 [43], 477 [76], and 503 [73] CpGs previously reported, in total ten overlap in two different studies; however, six of these are reported with opposite directions of association with AD [43, 73, 76]. Thus, only four CpGs had the same direction of association in AD between different studies [43, 73, 76]. These were cg08787968 and cg01693350 (both in the gene body of the *WT1* Transcription Factor), cg03294458 (in the gene body of Lysine Deficient Protein Kinase 4 - *WNK4*) [43, 76] and cg02768721 (in the gene body of Protein Tyrosine Phosphatase Receptor Type N2 - *PTPRN2*) [43, 73]. Even when comparing a thousand differentially methylated sites pre- and post-diagnosis in the same study (Supplementary Table 15), the number of overlapping sites is small, totaling only 7 CpGs with concordant directions [43].

## DISCUSSION

The present study aimed at leveraging a unique longitudinal design with up to 16 years of prospective pre-diagnosis data from an age- and sex-matched case-control sample of clinical AD to test the hypothesis that EAA measures in blood are predictive of AD. A secondary aim was to identify new potentially predictive or diagnostic CpGs using novel longitudinal and machine learning methods. No evidence was obtained for EAA being predictive of AD in our longitudinal cohort, or a cross-sectional validation sample. Our longitudinal AD panel was the only novel biomarker identified in Betula that replicated in AIBL, although with negligible discriminatory value.

The absence of evidence for blood-based EAA measures as biomarkers of AD conversion is consistent with several recent studies on pre-symptomatic dementia cases [77, 78], MCI, or manifest dementia cases [79], as well as observed null associations with Aβ, p-tau, or t-tau status in cerebrospinal fluid [36]. Similarly, a previous AIBL study reported largely null findings between age-acceleration and cross-sectional and longitudinal measures of neuroimaging, cognition, and Aβ load; except for a robust cross-sectional association between the Hannum clock and hippocampal volume in cognitively unimpaired individuals with high brain Aβ load [35]. In contrast, a previous small study from our group suggested an association between DNAm age and dementia status in Betula (*n* = 11, [34]), although lifestyle factors possibly associated with both AD and EAA [80] were not accounted for in that study. Further, the DunedinPACE, was recently shown to predict AD status in the two cohorts [41]. It is unclear why the present analyses did not replicate this association, particularly in AIBL, which should have adequate statistical power to detect the relatively modest effect size reported in that study (0.28 SD units). Future studies are needed to explore potential cohort-specific factors that influence predictive power of EEA for AD. However, for EAA measures to hold practical value for prediction, it is critical that they replicate across different cohorts and contexts and show added predictive ability over and above easily available clinical and lifestyle risk factors. Thus, currently the clinical utility of EAA is limited, and the present study extends previous findings by observing similar rates of EAA in cases and controls across 16 years prior to AD onset. It should be noted that in our study, as in previous ones, the correlations between epigenetic ages (Horvath, Hannum, PhenoAge) and chronological age were consistently high across both AD cases and controls [71], demonstrating the validity of our data and epigenetic age estimations in general. The possibility also remains that EAA in brain tissue significantly differentiates AD from controls [81], albeit not useful as a predictive biomarker due to its invasiveness; or that novel computational solutions improve the reliability of epigenetic clocks and thereby their predictive potential [94].

In addition to our null findings for the epigenetic clocks, our attempts to identify novel panels/scores of DNAm biomarkers with predictive value for AD had limited success. Given the scarcity of replication evidenced in the literature, it is noteworthy that our longitudinal AD panel consisting of 73 CpG sites replicated nominally in the AIBL validation sample. This despite differences in sample characteristics, such as diagnostic criteria, sex, and *APOE* *ɛ*4-proportions. However, the panel still lacks practical usefulness due to its low discriminatory accuracy in the external validation sample. We further explored replication of individual sites within this panel, and found that three of the 73 CpGs located in genes *ICOS*, *RBBP6*, and *POLE* replicated nominally in AIBL, all hypomethylated in AD cases across both cohorts. ICOS may have a role in the brain’s inflammation response by CD4+ and CD8+ T cells [82], RBBP6 participates in the regulation of inflammatory and immune processes through activation of the NF-*κ*B pathway [83], while POLE directly participates in the DNA repair [84]. Inflammation pathways play a known but still unclear role in increasing risk of AD [85–87], and have been highlighted in previous studies on blood-based DNAm in AD [10, 43, 73] and other neurodegenerative conditions [53].

A limited replication of CpGs was further observed in our literature review of blood-based DNAm array studies of AD with published lists of CpGs. This literature review showed that only 4 out of 3,275 identified CpGs replicated with concordant direction of effects across the studies [43, 73, 76]. These four CpGs were located in genes that participate in macrophage and immune responses, WNK4 and PTPN2 [88, 89], and the synaptic plasticity-related gene WT1 (2 CpGs) [90], respectively. The literature review did not identify previously replicated findings from candidate-gene or array-based DNAm studies, such as *BIN1*, *PIN1*, and *BDNF*, but instead it is in line with the low CpG replication rate reported for array-based studies in a previous review [8]. Thus, although limited in number, the replicated CpGs could be etiologically relevant for AD, and other studies have also convincingly identified several CpG sites with a potential mechanistic role in the disease [10]. However, the scarcity of CpG-level replication across studies is a challenge for developing robust DNAm biomarkers in blood. Particularly ones that hold predictive value over and above known well-known predictors like age, sex, or *APOE* *ɛ*4 in external samples. Recent attempts at developing more robust DNAm biomarkers through principal-component level rather than CpG-level training may be one way forward [94], as could targeting region-level methylation instead of CpG-level [44, 73]. The latter could be particularly valuable when whole-genome methylation data becomes more accessible, since array resolution is a limiting factor for identifying differentially methylated regions.

Many factors, in addition to methodological and analytical ones [94, 95] may contribute to the limited replication of blood-based DNAm EWAS findings in AD. The heterogeneity of the disease itself may also be a main contributor, as AD comprises several subtypes concerning genetic factors, neuropathology, cognitive symptoms, and biological pathways [7, 59, 96, 97]. Different study cohorts may differ on important disease characteristics potentially influencing DNAm. For instance, the Betula and AIBL samples differed in *APOE* *ɛ*4 carriage among AD cases (54% versus 77%), the number of males (18% versus 43%), as well as educational attainment (Table 1)— all factors which may influence DNAm patterns. One potential future direction for studies with larger sample sizes could be to test DNAm biomarkers in individuals with lower genetic AD risk, e.g., *APOE* *ɛ*4 non-carriers who still make up a sizable proportion of diagnosed AD cases, and for whom environmental AD risk factors captured by DNAm patterns [80] may play a larger role in disease development. Heterogeneity in DNAm alterations may also characterize different AD stages, i.e., early, pre-clinical stages, compared to intermediate, or late AD (see e.g., [98] for similar reasoning/findings on proteomic biomarkers). This was observed in both our non-overlapping pre- and post-AD onset sPLS results, and previous shorter (3–4.5 years) follow-up studies of pre- and post AD (7/1000 overlapping CpGs; [43]) and pre- and post all-cause dementia onset (4% overlap; [44]). However, potential subtype or stage-specific DNAm patterns still remain to be systematically investigated and replicated.

With regards to findings from DNAm studies, it is also relevant to consider the magnitude of the methylation differences observed. For instance, the mean methylation differences between Betula cases and controls after AD onset ranged from 4.7 to 8.2%, when considering the three validated CpGs of our longitudinal AD panel. This can be compared with a previous study in monozygotic and dizygotic AD-discordant twins with median methylation differences of 18.4% (min 15.9, max 29.7%) in blood examined by a different DNAm analysis method [99]. In previous studies in blood the median |Δ β| between AD cases and controls ranged from 1.0% to 4.6% (min 0, max 19.3%), indicating that methylation differences <5% are commonly reported [43, 72, 73, 76, 92]. It is still unknown if methylation changes in this range lead to biologically significant changes in gene expression, but some evidence indicates biological relevance of subtle DNAm changes, for instance by resulting in protein isoform diversity [100]. Furthermore, in studies based on several cell types, small average changes in methylation levels may mask larger underlying changes in specific cell types. Regardless of functional consequences, small methylation differences could still provide valuable information as indicators rather than causes of dysregulated biological pathways, or serve a predictive role regardless of their functional relevance, as exemplified by the CpGs included in the epigenetic clocks that successfully predict other age-related disorders and mortality.

We note that within our longitudinal AD panel the majority of CpGs (78%), and all three validated CpGs, were cross-sectionally hypomethylated in AD cases compared to controls at the time of diagnosis. This is concordant with some recent studies on blood DNAm [10, 44, 76, 101] but not with others, that instead observed hypermethylation [43, 72, 73, 92]. Also longitudinally, almost all sites where DNAm rate of change significantly differed between cases and controls (i.e., time-by-AD interaction) in our longitudinal panel evidenced decreased methylation over time in AD. A decrease in global methylation levels with aging has been seen in several tissues [102], and thus it is possible that DNAm changes follow the same direction in aging as in AD, as previously proposed [10, 33].

Our current findings may have methodological and study design implications. Firstly, we applied a novel multivariate method, sPLS-DA, to try to identify CpGs in any parts of the genome that could jointly differentiate AD cases from controls. Although novel machine-learning or artificial intelligence methods are emerging for DNAm analyses [72], the majority of AD studies so far have relied on univariate methods (or differentially methylated regions across adjacent CpGs). In our data, the generated pre- and post-AD scores that were estimated based on sPLS-DA in the Betula sample were not replicated in AIBL. sPLS-DA is considered to be able to outperform other machine learning methods of feature selection due to its sparsity assumption [68], that aims at reducing the number of features that conjunctly discriminate the analyzed condition. This would help avoiding the selection of “noise” variables [103]. Even so, overfitting does happen [103], reinforcing the need for internal and external validation of the selected features. There was low concordance in the identified CpGs between the two different methods used in the current study, LMMs for the longitudinal AD panel and sPLS-DA for the pre- and post-AD scores. The fact that out-of-sample replication was seen only for the longitudinal panel, comprising stably differentially methylated sites between cases and controls across up to 26 years of follow-up, may speak to the superiority of longitudinal study designs for identifying DNAm-based disease biomarkers. This may be particularly true for small sample sizes such as in the current study, where the repeated measurements may act as intra-individual replication aiding identification of more reliable CpGs.

A strength of our study is that we considered estimated blood cell proportions in our analyses, which may otherwise confound DNAm estimates if differentially affected by the health status or age of the study participants [62, 104]. We also separately analyzed potential longitudinal differences in estimated blood cell counts in AD cases and controls. An increase in NK cells proportion over time was seen only in AD cases, again indicating a potential change in AD inflammation/immune response with aging. To the best of our knowledge, this is the first study of longitudinal changes in blood cell compositions in AD, and additional studies are needed to consolidate this finding. We also replicated some previous age-related changes in blood cell counts, like a decrease in subtypes of CD4+ T cells and B cells [105], and increase in monocytes [106].

### Limitations

The limited AD sample size in the Betula study is an important limitation of our study, but uniqueness of the dataset with longitudinal retrospective blood samples up to 16 years prior to diagnosis nevertheless had potential to bring novel insights into the long-term predictive ability and temporal dynamics of blood-based DNAm biomarkers for AD. All available AD cases in the Betula study database who fulfilled the inclusion and exclusion criteria were included. Still, we acknowledge that this study was likely underpowered, particularly for the EWAS analyses, and lack of correction for multiple comparisons may have increased risk of false positive results. Another limitation was the lack neuropathological data or gold standard biomarkers (cerebrospinal fluid or positron emission tomography neuroimaging) to confirm AD diagnoses, which were not available in the Betula study, our primary cohort. Even so, the diagnostic evaluation integrated health-related, clinical, and cognitive assessments, resulting in a reliable clinical characterization [37, 45]. An inherent limitation for epigenetic clocks is the underestimation of epigenetic age in older subjects, which can lead to a loss of precision in older samples [70]. Finally, it is important to stress that the EPIC array covers only a small fraction, 850,000 of the ∼28 million CpG sites in the genome [107] and CpGs associated with AD could be outside the currently analyzed regions. Whole genome bisulfite sequencing has the potential to identify novel disease-associated CpGs.

### Conclusions

The findings of this 16-year longitudinal study concur with the majority of recent observations in the literature that blood-based EAA measures developed so far are of limited value as AD biomarkers, particularly when other easily available indicators such as age, sex, and blood cell proportions are accounted for. Tentatively, inflammation and immune-system related processed may be reflected in DNAm patterns in AD, but overall our findings underscore the difficulty of identifying replicable epigenome-wide DNAm alterations that can reliably distinguish AD cases from controls beyond known markers such as *APOE* *ɛ*4 genotype.

## Supplementary Material

Supplementary Material

## Data Availability

The Betula dataset used and/or analyzed in the current study is available from the corresponding author on reasonable request, as long as the data transfer is in agreement with the European Union legislation on the General Data Protection Regulation and Umeå University data protection policies. AIBL DNAm data are available from the GEO repository accession number GSE153712.
